# Supplementary material for: Tumor Immune Microenvironment and Its Clinicopathological and Prognostic Associations in Canine Splenic Hemangiosarcoma
Source: Animals (Basel). 2024 Apr 18;14(8):1224. doi: 10.3390/ani14081224 (PMC11047608; doi:10.3390/ani14081224)
Supplement: Supplementary file 1 [file animals-14-01224-s001.zip › animals-2924012-supplementary.pdf]

Supplementary Table S1. Signalment and follow-up data of the animals included in the study.

Dog N.	Breed	Gender	Age	Stage*	Metastasis	Outcome	Overall survival (days)
1	Labrador retriever	F	10	n.a.	N	Unfavourable	229
2	German shepherd	F	13	I	N	Favourable	723
3	German shepherd	M	10	II	N	Unfavourable	21
4	German shepherd	M	11	n.a.	n.a.	n.a.	n.a.
5	Belgian shepherd	M	8	n.a.	n.a.	n.a.	n.a.
6	Springer spaniel	M	8	n.a.	n.a.	n.a.	n.a.
7	n.a.	M	12	II	Y	Unfavourable	46
8	n.a.	M	9	II	n.a.	Favourable	971
9	Australian Shepherd	M	9	n.a.	n.a.	n.a.	n.a.
10	Mixed breed	M	9	n.a.	n.a.	n.a.	n.a.
11	Labrador retriever	F	12	n.a.	n.a.	n.a.	n.a.
12	Cane Corso	M	10	n.a.	n.a.	n.a.	n.a.
13	Boxer	F	8	II	N	Unfavourable	n.a.
14	Mixed breed	M	13	III	Y	Unfavourable	113
15	German shepherd	FS	10	II	N	Unfavourable	n.a.
16	Pomeranian	M	n.a.	n.a.	n.a.	Unfavourable	n.a.
17	Australian Shepherd	F	n.a.	n.a.	n.a.	n.a.	n.a.
18	Mixed breed	M	9	III	Y	Unfavourable	91
19	Ariegeois	FS	10	II	Y	Unfavourable	145
20	Labrador retriever	M	n.a.	n.a.	n.a.	n.a.	n.a.
21	n.a.	F	12	II	N	Favourable	611
22	Mixed breed	M	16	III	Y	Unfavourable	164
23	Epagneul Breton	F	10	II	Y	Unfavourable	509
24	Golden retriever	FS	12	III	Y	Unfavourable	28
25	Mixed breed	M	12	III	Y	Unfavourable	27
26	Jack russel terrier	F	n.a.	II	Y	Unfavourable	395
27	Jack russel terrier	M	14	n.a.	n.a.	n.a.	n.a.
28	Mixed breed	F	13	II	Y	Unfavourable	72

29	Mixed breed	F	12	I	Y	Unfavourable	137
30	Labrador retriever	M	4	II	n.a.	Favourable	452
31	Mixed breed	M	10	II	n.a.	Unfavourable	52
32	Beagle	M	11	II	Y	Unfavourable	245
33	Mixed breed	FS	8	n.a.	n.a.	Favourable	362
34	German shepherd	n.a.	10	III	Y	Unfavourable	44
35	Labrador retriever	F	11	III	Y	Unfavourable	106
36	Mixed breed	M	13	I	Y	Unfavourable	97
37	American staffordshire terrier	MC	9	II		Unfavourable	n.a.
38	Mixed breed	F	10	III	Y	Unfavourable	109
39	Mixed breed	FS	12	III	Y	Unfavourable	67
40	Labrador retriever	M	10	n.a.	n.a.	n.a.	n.a.
41	Labrador retriever	M	9	n.a.	Y	Unfavourable	37
42	Mixed breed	M	13	I	Y	Unfavourable	230
43	French Bouledogue	M	6	I	N	Unfavourable	535
44	Poodle	FS	12	II	n.a.	Unfavourable	n.a.
45	Labrador retriever	MC	10	II	Y	Unfavourable	10
46	Cocker Spaniel	M	11	II	Y	Unfavourable	199
47	Vizla	M	11	I	N	Unfavourable	9
48	Boxer	MC	12	III	Y	Unfavourable	311
49	Jack russel terrier	M	11	I	N	Favourable	485
50	Boxer	M	8	III	Y	Unfavourable	183
51	German shepherd	FS	13	II	Y	Unfavourable	296
52	Mixed breed	FS	14	III	Y	Unfavourable	128
53	German shepherd	M	12	n.a.	N	Unfavourable	n.a.
54	Mixed breed	FS	12	II	Y	Unfavourable	102
55	Mixed breed	M	7	II	Y	Unfavourable	n.a.
56	Mixed breed	M	14	II	n.a.	Favourable	81

*Staging system according to Withrow et al [4]. n.a.= not available

Supplementary Table S2. Immunohistochemical data of the animals included in the study.

Dog N.	Iba-1		FoxP3		CTLA-4		CD3			CD20		
	Distribution	Quantity	Distribution	Quantity	Distribution	Quantity	Distribution	Quantity	Groups	Distribution	Quantity	Groups
1	2	5.6	1	1	0	0	2	4.5	0	2	4.6	0
2	0	0	0	0	0	0	2	10.8	0	1	6.8	0
3	3	42.4	2	2.9	2	0.2	1	13.4	0	1	3.2	0
4	2	10	2	2.8	0	0	2	24.2	2	2	40.2	0
5	1	0.4	0	0	2	0.4	1	5	0	0	2.7	0
6	2	10.4	2	12.5	0	0	2	66.1	2	2	51.4	2
7	3	65	2	3.7	2	0.5	3	48.9	2	3	37.8	2
8	3	55.8	0	0	2	0.5	2	24.3	0	0	1	0
9	2	13.1	2	2.2	2	1.2	2	6.9	0	0	2	0
10	1	3	2	3.4	0	0	3	19.6	0	1	2.8	0
11	3	81	2	1.2	0	0	2	37.4	0	2	34.1	1
12	2	5.6	2	7.4	2	2.7	3	39.8	2	2	20.3	1
13	2	29.4	2	5.5	0	0	3	62	2	1	7.4	1
14	0	0	2	2.4	2	3.1	3	42.8	0	2	20.7	1
15	2	2.9	1	0.2	1	0.2	2	3.8	0	0	0.6	0
16	3	42.6	2	7.3	3	4.5	3	93.5	0	2	44.4	2
17	2	11	2	5.3	2	3.8	1	16.8	0	1	8.5	0
18	0	0	2	0.4	2	0.8	2	45.1	0	1	23.6	0
19	1	3.4	2	1.4	2	0.2	3	18.2	0	0	1.7	0
20	2	2.6	2	2.7	1	0.2	3	97.5	2	2	25.6	1
21	2	55.1	2	3	0	0	2	33.8	0	1	4.6	0
22	2	6	2	4.2	2	3.3	3	96.8	2	2	42.2	1
23	0	0	0	0	0	0	1	8.4	0	2	32.5	0
24	0	0	2	2.9	0	0	2	11.2	0	2	7.7	0
25	3	35.8	2	0.5	0	0	2	15.2	0	0	0.6	0
26	2	5.1	2	1.8	0	0	2	16.5	0	2	17.2	0

27	3	11.2	2	0.7	0	0	1	4.8	0	0	3.1	0
28	2	3.3	2	6.9	2	1	2	53.2	2	1	4	0
29	2	3.8	2	3	0	0	1	11.6	0	0	0.4	0
30	3	119.2	2	8.3	2	2.4	2	54.4	2	3	45.4	1
31	2	14.3	2	4.5	2	1.2	2	62.9	2	2	49.3	2
32	2	19.2	2	3.1	2	1	1	19.4	0	0	0.4	0
33	2	17.2	2	8.9	2	1.1	3	14.1	0	2	19	1
34	1	6	2	23.9	1	0.2	3	89.4	0	0	1.2	0
35	3	48.6	2	1.7	2	2.2	3	38.5	1	3	11.2	0
36	2	22.7	2	6.9	2	1.2	2	4.5	0	2	29.7	2
37	1	5	2	1.9	1	0.2	3	78	2	0	1.4	0
38	3	60.3	2	10	1	0.6	3	98.5	2	1	25.7	2
39	2	20.7	2	0.8	0	0	3	39.2	0	2	34.8	1
40	2	31.2	2	1.4	0	0	2	16.8	0	0	1.2	0
41	0	0	2	2	0	0	3	n.a.		3	73.6	2
42	2	2.4	1	1	0	0	2	48.5	2	1	0.2	0
43	3	143.8	1	1	3	4	2	76.3	2	2	76.4	2
44	2	6.4	2	1.3	0	0	1	6	1	2	21.9	1
45	2	37.2	1	0.2	1	0.2	1	39.1	1	2	6	0
46	3	35.2	2	5.3	3	4.2	2	6.5	0	2	21.5	2
47	2	13.5	1	0.8	0	0	3	56.6	0	1	4.7	0
48	3	43.6	1	2.2	2	1	3	30.7	2	2	42.7	0
49	2	8.9	3	17.8	3	5.1	2	30.7	1	2	5.3	0
50	2	23.6	2	1.9	2	0.4	2	14.4	1	1	9	1
51	2	2.4	3	8.8	2	3.2	3	62.7	2	2	56.2	2
52	2	20.6	2	2.4	3	4.9	1	55.4	2	1	21.8	2
53	2	3.5	1	0.4	2	1	2	6.9	0	2	2.9	0
54	2	1.8	2	3.3	2	3.8	2	7	0	1	0.8	0
55	3	62.9	1	0.8	2	1.1	1	13.1	1	2	69.4	2
56	2	10.3	1	1.2	2	1.1	1	49	2	2	0.6	0

Distribution: 0 = absent, 1 = focal, 2 = multifocal, 3 = diffuse.
Quantity: average number of positive cells per unit area (0.237 mm^2).
Groups: 0 = single cells, 1 = small aggregates, 2 = massive aggregates (≥ 30 cells).
n.a.= not available
